# Early-Life Enteric Pathogen Exposure, Socioeconomic Status, and School-Age Cognitive Outcomes

**DOI:** 10.4269/ajtmh.22-0584

**Published:** 2023-08-02

**Authors:** Rebecca J. Scharf, Elizabeth T. Rogawski McQuade, Erling Svensen, Amber Huggins, Angelina Maphula, Eliwaza Bayo, Ladislaus Blacy, Paula Pamplona E. de Souza, Hilda Costa, Eric R. Houpt, Pascal O. Bessong, Estomih Mduma, Aldo A. M. Lima, Richard L. Guerrant

**Affiliations:** ^1^ Department of Pediatrics, Neurology and Public Health, University of Virginia, Charlottesville, Virginia;; ^2^ Department of Epidemiology, Emory University, Atlanta;; ^3^ Department of Organizational Psychology, Haukeland University Hospital, Bergen, Norway;; ^4^ Department of Public Health, University of Virginia, Charlottesville, Virginia;; ^5^ Department of Psychology, University of Venda, Thohoyandou, South Africa;; ^6^ Haydom Research Center, Haydom, Tanzania;; ^7^ Department of Psychology, Federal University of Ceará, Fortaleza, Brazil;; ^8^ Department of Medicine, University of Virginia, Charlottesville, Virginia;; ^9^ Department of Microbiology, Federal University of Ceará, Fortaleza, Brazil

## Abstract

Early-life experiences of enteric infections and diarrheal illness are common in low-resource settings and are hypothesized to affect child development. However, longer-term associations of enteric infections with school-age cognitive outcomes are difficult to estimate due to lack of long-term studies. The objective of this study was to examine the relationship between enteropathogen exposure in the first 2 years of life with school-age cognitive skills in a cohort of children followed from birth until 6 to 8 years in low-resource settings in Brazil, Tanzania, and South Africa. The study included participants from three sites from the Etiology, Risk Factors, and Interactions of Enteric Infections and Malnutrition and the Consequences for Child Health Study who were enrolled just after birth and followed for enteric infections, diarrheal illness, and cognitive development until 2 years of age. When the children were school-age, further data were collected on reasoning skills and semantic/phonemic fluency. We estimated associations between the burden of specific enteric pathogens and etiology-specific diarrhea from 0 to 2 years with cognitive test scores at 6 to 8 years using linear regression and adjusting for confounding variables. In this study, children who carried more enteric pathogens in the first 2 years of life showed overall decreases in school-age cognitive abilities, particularly children who carried protozoa, although this was not statistically significant in this sample. Socioeconomic factors such as maternal education and income were more closely associated with school-age cognitive abilities. Early-life enteric pathogens may have a small, lasting influence on school-age cognitive outcomes, although other socioeconomic factors likely contribute more significantly.

## INTRODUCTION

Enteric infections are common among children in low- and middle-income countries.^1^ In addition to causing diarrheal illness, enteric infections contribute to environmental enteropathy and have been associated with malnutrition, growth shortfalls, school days lost due to poor health, and challenges to developmental progress in children.^2^^,^^3^ Importantly, even in the absence of diarrhea, high burdens of enteric pathogens may have impacts on children as they grow.^4^^,^^5^ For example, intestinal bacterial burden in nondiarrheal stools and burden of specific pathogens, including *Shigella*, *Campylobacter*, *Giardia*, and enteroaggregative *Escherichia coli*, negatively correlated with anthropometric measures at 2 and 5 years of age in the Etiology, Risk Factors, and Interactions of Enteric Infections and Malnutrition and the Consequences for Child Health (MAL-ED) birth cohort study.^5^^,^^6^

Although early-life anthropometric measures are associated with cognitive outcomes^7^ and effects on anthropometry are often assumed to correspond to effects on cognition, few studies are able to follow children from birth through school years to assess associations directly between early-life exposures and cognitive/academic outcomes. School-age performance is an important predictor of future independence and contributions to communities.

The MAL-ED is a prospective cohort study that aims to understand the interconnected relationship between malnutrition and enteric disease, and how they affect the development of children in low-resource settings. Children from the MAL-ED Study were found to have increased fecal biomarkers for intestinal inflammation, systemic inflammation, and/or intestinal permeability.^8^^,^^9^ In addition, the burden of enteropathogens and days with illness negatively correlated with cognitive outcomes measured at the age of 2 in MAL-ED.^10^ However, the long-term impact of early-life infections on cognitive development is unknown. In this study, we measured reasoning skills and verbal fluency in children in three of the MAL-ED sites at age 6-8 years to assess whether enteric disease early in life and socioeconomic status were associated with school-aged cognitive outcomes.

## MATERIALS AND METHODS

The overall study design of the MAL-ED study,^11^ participant baseline characteristics,^12^^–^^14^ and primary outcomes,^15^ have been described elsewhere. To summarize, eight sites with high rates of malnutrition and enteric disease were chosen, and children were followed from birth to 24 months of life. Information was collected on enteric pathogens, nutrition, sanitation, socioeconomic status, growth and cognitive outcomes. For this study, in three of the participating sites, further cognitive data were collected when the children were 6 to 8 years of age. In this study, we report the relationship between early enteropathogen infections, diarrheal illnesses, socioeconomic status, and cognitive development of 8-year-old children in the MAL-ED cohort.

### Predictors.

Nondiarrheal stool samples were collected monthly from the children enrolled in the study from 1 to 24 months of age. These samples were analyzed for 29 enteric pathogens using quantitative polymerase chain reaction (qPCR), as previously described.^16^ Total pathogen burden was quantified as the average number of pathogens detected in nondiarrheal stools, as well as the total bacterial, viral, and parasitic infection burden. Burden of specific pathogens was calculated as the percent of nondiarrheal stools positive for each pathogen (including the 13 most common pathogens in nondiarrheal stools: *Cryptosporidium*, *Campylobacter*, *Giardia*, *Shigella*, enteroaggregative *E. coli*, typical enteropathogenic *E. coli* (EPEC), atypical EPEC, heat-labile enterotoxigenic *E. coli* (ETEC), heat-stable (ETEC), norovirus GII, adenovirus 40/41, astrovirus, and sapovirus, as previously described.^5^ Samples were also collected and tested by qPCR during diarrhea episodes, and diarrhea etiologies were assigned based on the quantity of pathogen detected and the association between pathogens and diarrhea, previously analyzed.^17^ Briefly, pathogen-attributable fractions were calculated for each episode based on the odds ratios from a mixed effects model associating pathogen quantity with diarrhea. Episodes with pathogen-specific attributable fractions > 0.5 (i.e., majority attribution) were attributed to that pathogen.^5^ Infectious diarrhea was defined if any pathogen could be attributed to the episode.

### Covariates.

In this study, a measure of socioeconomic status was calculated based on the family’s access to clean water, household assets, maternal education, and income.^18^ We also examined predictors and outcomes by child’s age, gender, research site, maternal education, enrollment weight (proxy for birthweight), maternal height, and breastfeeding status. These variables were measured in infancy.

### Outcomes.

The Raven’s Colored Progressive Matrices^19^^,^^20^ (RCPM) is a reasoning assessment developed to assess reasoning skills across the lifespan. In the MAL-ED study, this assessment was used to assess mothers of the participating children when the children were between 6 and 8 months of age.^21^ The RCPM is a visual, non–language-based assessment that tests the participant’s ability to solve a series of puzzles, set up in analogy form, to form comparisons. The test uses shapes and designs rather than language or pictures, and thus it is thought to be less culture or language specific. The items become progressively more challenging, allowing for assessment of reasoning along a continuum of puzzles. This assessment has been used before in populations that may not have access to formal schooling.^20^ Because the research sites have experience with this measure, and it remained appropriate for children who may not have attended school and is language-independent, we chose to use the RCPM in the 6- to 8-year-old children as well. The assessment was piloted in all three sites on children not participating in the MAL-ED study and found to be acceptable to the children and provide a variety of scores. The assessment was then administered to children from the MAL-ED study by trained assessors in as quiet and distraction-free location as available. Children were shown example problems and asked to point to the correct answer. When a child had five incorrect answers in a row, the assessment was stopped.

Next, verbal fluency was assessed in the children, using a brief assessment of a subtest from the NEPSY Neuropsychological Exam.^22^ Semantic fluency was assessed by asking the children to name as many animals as they could in 1 minute and then to name as many foods as they could in 1 minute. Phonemic fluency was assessed by asking children to name as many words that began with S they could in 1 minute and then to name as many words that begin with F as they could in 1 minute. This assessment was chosen because it is a brief, simple assessment that has been used at our sites in the past. In addition, there is previous evidence that childhood diarrhea burden may have associations with verbal fluency.^23^ This assessment was piloted on children outside the study in all three sites using foods, animals, and words that start with S, N, F, and P. In all languages, children generated more words beginning with S than F (indicating progressing difficulty) and there were relevant words in all languages. z-scores were created for each score, by site, to allow for comparison of child’s performance relative to peers from their site, across sites. One z-score is a single standard deviation difference based on the standard deviation of scores observed in our study population.

### Analysis.

Variables were examined and assessed for normality. First, we estimated the associations between total pathogen burden scores in nondiarrheal stool samples for all pathogens, bacteria, viruses, and protozoa with semantic fluency, phonemic fluency, and reasoning skills scores using linear regression, and adjusting for site, sex, maternal education, enrollment weight-for-age z-score, percent days exclusively breastfed in the first 6 months of life, maternal height, socioeconomic status, and age at cognitive outcome assessment. Effect sizes are interpreted per additional pathogen detected on average in nondiarrheal stools from 1 to 24 months of age. Next, the associations between then burden of specific pathogens in nondiarrheal stools and the three cognitive outcomes were estimated using similar models that were additionally adjusted for the other pathogens. Effects were scaled to compare a high burden of pathogen (at the site-specific 90th percentile of stool positivity between 1 and 24 months) to a low burden of pathogen (at the 10th percentile of stool positivity between 1 and 24 months), as previously.^5^ Scores were reported as change in z-score, with 95% CIs and statistical significance (*P* values) reported as well.

We next estimated the associations among all-cause, infectious, class-specific, and etiology-specific diarrhea and the cognitive outcomes using linear regression and adjusting for the same covariates listed earlier. Associations with all-cause and infectious diarrhea episodes are scaled per additional episode experienced from birth to 2 years of age. Because few children had multiple etiology-specific episodes, for specific etiologies, we compared children who had at least one episode to children who had no episodes attributed to that pathogen. Finally, we estimated the associations between baseline sociodemographic characteristics and the three cognitive outcomes using multivariable linear regression.

## RESULTS

Table 1 shows demographics of the study population in total and by study site. In general, children at the study site in Fortaleza, Brazil were assessed at a younger age than the children in the Venda, South Africa and Haydom, Tanzania (TZH) research sites and thus age was included as a variable in all statistical models. Socioeconomic status (measured through access to clean water, family assets, maternal education, and family income) was generally lowest in the TZH site.

**Table 1:** Study population characteristics

Parameter	Median (IQR)			
	Fortaleza, Brazil (*N* = 117)	Venda, South Africa (*N* = 171)	Haydom, Tanzania (*N* = 163)	All (*N* = 451)
Sociodemographic characteristics				
Age at 6- to 8-year assessment	6.85 (6.44 to 7.24)	8.01 (7.05 to 8.16)	7.70 (7.33 to 8.02)	7.49 (7.04 to 8.02)
Age at 24-month assessment	2.03 (2.0 to 2.04)	2.01 (1.98 to 2.03)	2.01 (1.97 to 2.16)	2.02 (1.99 to 2.04)
Female sex, *n* (%)	53 (45.3)	84 (49.12)	82 (50.31)	219 (48.56)
Male sex, *n* (%)	64 (54.7)	87 (50.88)	81 (49.69)	232 (51.44)
% days exclusively breastfed < 6 months	0.49 (0.28 to 0.71)	0.17 (0.1 to 0.29)	0.32 (0.19 to 0.44)	0.28 (0.15 to 0.46)
Mean WAMI* score	0.83 (0.77 to 0.90)	0.8 (0.72 to 0.85)	0.21 (0.14 to 0.28)	0.72 (0.27 to 0.84)
Maternal height (cm)	155 (150 to 160)	158 (155 to 162)	156.5 (152 to 160)	157 (152 to 161)
Maternal education (years)	9 (7 to 12)	11 (9 to 12)	7 (3 to 7)	8 (7 to 11)
Improved water, *n* (%)	117 (100)	157 (92.35)	103 (63.19)	377 (83.78)
Improved sanitation, *n* (%)	117 (100)	167 (98.24)	10 (6.1)	294 (65.33)
Monthly income (U.S. dollars)	345.81 (292.13 to 412.81)	246.51 (164.37 to 396.84)	21.0 (13.17 to 39.0)	186 (32.56 to 344.93)
No. of diarrheal episodes 0–24 months	1 (0 to 2)	1 (0 to 2)	2 (1 to 4)	1 (0 to 2)
Anthropometry				
Enrollment WAZ	−0.17 (−0.77 to 0.47)	−0.40 (−0.96 to 0.14)	−0.02 (−0.61 to 0.64)	−0.20 (−0.83 to 0.43)
2-year LAZ	−0.03 (−0.87 to 0.73)	−1.62 (−2.46 to −1.05)	−2.59 (−3.22 to −1.98)	−1.73 (−2.60 to −0.74)
5-year HAZ	−0.08 (−0.76 to 0.38)	−1.05 (−1.56 to −0.40)	−1.93 (−2.53 to −1.24)	−1.15 (−1.92 to −0.36)
6- to 8-year HAZ	0.13 (−0.74 to 0.54)	0.14 (−0.82 to 0.52)	−1.56 (−2.15 to −1.03)	−0.65 (−1.56 to 0.15)

HAZ = height-for-age z-score; IQR = interquartile range; LAZ = weight-for-age z-score; WAMI = Water/Sanitation, Assets, Maternal education, and Income; WAZ = weight-for-age z-score.

* WAMI is a measure of socioeconomic status that includes family’s access to clean water, household assets, maternal education, and income.

**Figure 1.**
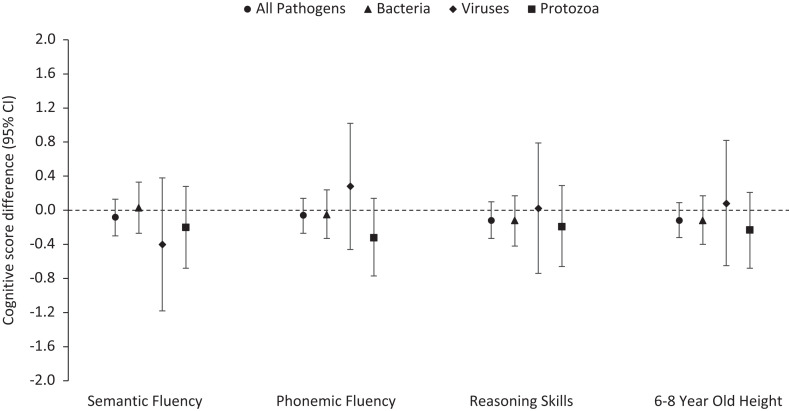
Associations between early enteric pathogen infections and cognitive outcomes and growth at school-age. Effect of one additional pathogen detected on average from 0 to 24 months of age. Adjusted for percent exclusive breastfed, age, enrollment weight (birthweight proxy), socioeconomic status, maternal height, maternal education, sex, and research site.

In general, larger enteric pathogen burdens were associated with lower cognitive scores (Figure 1), even when controlling for site, breastfeeding status, sex, enrollment weight, socioeconomic status, maternal height, maternal education, and age, although the associations were not statistically significant. For example, an additional enteric pathogen detected on average in nondiarrheal stools in the first 2 years of life was associated with −0.12 z-scores (95% CI: −0.33 to 0.10) on the Raven assessment. Negative associations were observed for bacterial and protozoal pathogens but not viral pathogens. A higher burden of protozoa in nondiarrheal stool samples had the strongest association with reasoning skills (Raven z-score difference per additional protozoa detected on average in nondiarrheal stool samples: −0.19, 95% CI: −0.66 to 0.29) and semantic (z-score difference: −0.20, 95% CI: −0.68 to 0.28) and phonemic fluency (z-score difference: −0.32, 95% CI: −0.77 to 0.14). Similar negative associations were observed between enteric pathogens before 2 years of age and height-for-age z-score measured at 6 to 8 years.

**Figure 2.**
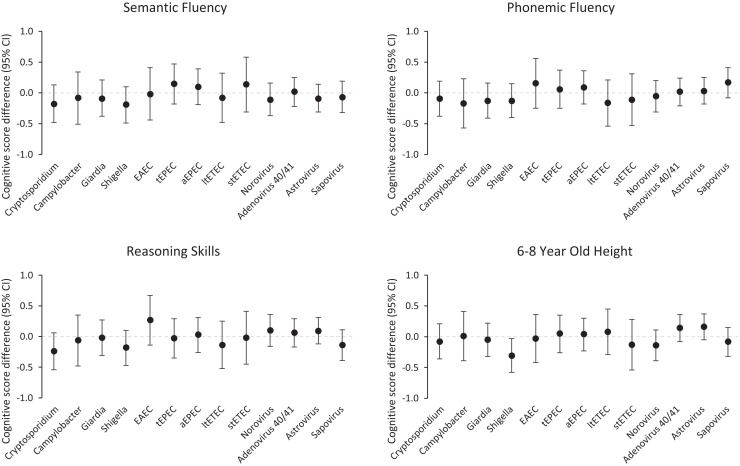
Associations between early specific enteric pathogen infections and cognitive outcomes at school-age. All models adjusted for percent exclusive breastfed, age, enrollment weight (birthweight proxy), socioeconomic status, maternal height, maternal education, sex, and research site.

Associations between the burden of specific enteric pathogens in the first 2 years of life and cognitive outcomes at school age were also generally small and not statistically significant (Figure 2). A high burden of *Cryptosporidum* and *Shigella* were associated with the largest decrements in cognitive skills. For example, a high burden of *Cryptosporidium* was associated with −0.24 Raven z-scores (95% CI: −0.54 to 0.06) compared with a low burden of *Cryptosporidium.* A high burden of *Shigella* was associated with a −0.18 z-score decrement (95% CI: −0.47 to 0.10) in semantic fluency. In a sensitivity analysis in which pathogen burden was defined by detections in both diarrheal and nondiarrheal stools, the results were unchanged.

The associations between etiology-specific diarrhea and 6- to 8-year-old cognitive outcomes were also relatively small (Table 2). In general, additional diarrheal episodes attributed to protozoal pathogens were associated with decrements in cognitive scores, although these estimates were imprecise (z-score difference per additional episode: −1.00, 95% CI: −2.39 to 0.39 for semantic fluency, −1.02, 95% CI: −2.34 to 0.30 for phonemic fluency, −1.11, 95% CI −2.47 to 0.26 for reasoning skills). Conversely, viral diarrhea episodes were more often associated with a positive increase in cognitive skills, although these estimates were not statistically significant (Table 2). Diarrhea episodes attributed to *Campylobacter jejuni/coli* and adenovirus 40/41 had the strongest negative associations with semantic and phonemic fluency. Norovirus GII and sapovirus diarrhea episodes had the strongest negative association with reasoning skills. In contrast to the associations with subclinical *Shigella* infections, *Shigella*-attributable diarrhea episodes were not associated with semantic fluency or reasoning skills and had a small negative association with phonemic fluency (z-score difference: −0.15, 95% CI: −0.54 to 0.23).

**Table 2:** Associations between etiology-specific diarrhea and school-age cognitive outcomes

Parameter	Estimate (CL) *P* value			
	Semantic fluency	Phonemic fluency	Reasoning skills	Height
	Words/animals in a minute from NEPSY	Words beginning with S & F in a minute from NEPSY	Raven colored progressive matrices	Measured during school-aged testing
Number of diarrhea episodes in 0–24 months				
Any episodes*	0.005 (−0.05, 0.06) 0.86	−0.001 (−0.05, 0.05) 0.96	−0.04 (−0.10, 0.01) 0.12	0.003 (−0.05, 0.06) 0.92
Any episodes attributable to a specific pathogen*	0.08 (−0.08, 0.23) 0.33	0.01 (−0.13, 0.16) 0.85	−0.06 (−0.20, 0.09) 0.46	0.03 (−0.12, 0.18) 0.70
Episodes attributable to bacteria*	−0.02 (−0.29, 0.26) 0.91	−0.06 (−0.32, 0.20) 0.66	0.09 (−0.18, 0.36) 0.50	0.07 (−0.20, 0.34) 0.61
Episodes attributable to viruses*	0.20 (−0.06, 0.46) 0.12	0.05 (−0.19, 0.30) 0.68	−0.25 (−0.50, 0.004) 0.05	−0.07 (−0.34, 0.19) 0.58
Episodes attributable to protozoa*	−1.00 (−2.39, 0.39) 0.16	−1.02 (−2.34, 0.30) 0.13	−1.11 (−2.47, 0.26) 0.11	−0.34 (−1.66, 0.99) 0.62
Comparison of children with ≥ 1 episode of diarrhea attributable to a specific pathogen with children with 0 episodes attributable to that pathogen				
*Campylobacter jejuni/coli*	−0.60 (−1.38, 0.17) 0.13	−0.23 (−0.97, 0.52) 0.55	0.17 (−0.56, 0.89) 0.65	−0.11 (−0.91, 0.69) 0.79
Sapovirus	0.52 (0.09, 0.97) 0.02	0.38 (−0.04, 0.80) 0.08	−0.19 (−0.63, 0.25) 0.40	−0.11 (−0.54, 0.33) 0.63
Enterotoxigenic *E. coli*	−0.26 (−0.70, 0.11) 0.16	0.09 (−0.30, 0.48) 0.64	0.05 (−0.46, 0.35) 0.80	0.11 (−0.30, 0.51) 0.61
Astrovirus	0.09 (−0.45, 0.63) 0.75	0.23 (−0.30, 0.75) 0.39	−0.13 (−0.67, 0.41) 0.64	−0.06 (−0.63, 0.51) 0.85
Norovirus GII	0.18 (−0.22, 0.62) 0.35	−0.04 (−0.45, 0.36) 0.83	−0.21 (−0.63, 0.21) 0.33	0.0004 (−0.43, 0.43) 1.00
Rotavirus	0.59 (0.12, 1.06) 0.01	0.16 (−0.29, 0.62) 0.48	0.04 (−0.43, 0.51) 0.85	−0.08 (−0.54, 0.39) 0.74
Adenovirus 40/41	−0.25 (−0.85, 0.35) 0.42	−0.37 (−0.95, 0.20) 0.20	−0.01 (−0.59, 0.57) 0.97	0.48 (−0.09, 1.06) 0.10
*Shigella*	−0.02 (−0.42, 0.39) 0.93	−0.15 (−0.54, 0.23) 0.44	0.04 (−0.36, 0.43) 0.86	0.09 (−0.31, 0.49) 0.66

CL = confidence limits. Adjusted for %exclusive breastfed, age, enrolment weight (birthweight proxy), socioeconomic status, maternal height, maternal education, sex and research site.

* Effects per additional episode attributed.

Compared with the associations between enteric pathogens and the school-age cognitive outcomes, sociodemographic variables were more strongly associated with the cognitive outcomes (Table 3). Maternal height and maternal education were positively related to semantic fluency and reasoning skills. Family income was significantly associated with all the cognitive outcomes measured. Height measurements at 2, 5, and 6 to 8 years of age were strongly associated with the cognitive outcomes. For example, a 1 unit increase in length-for-age z-score at age 2 years was associated with 0.17 z-score increase (95% CI: 0.08–0.27) in semantic fluency, a 0.17 z-score increase (95% CI: 0.08–0.26) in phonemic fluency, and a 0.14 z-score increase (95% CI: 0.05–0.24) in reasoning skills. Associations were slightly stronger between height at 6 to 8 years and cognition at the same age.

**Table 3:** Childhood factors associated with semantic fluency and reasoning

Parameter	Estimate (CL) *P* value		
	Semantic fluency	Phonemic fluency	Reasoning skills
	Words/animals in a minute from NEPSY	Words beginning with S & F in a minute from NEPSY	Raven colored progressive matrices
Age at assessment	0.002 (−0.19, 0.19) 0.98	0.43 (0.24, 0.62) <0.0001	0.14 (−0.05, 0.33) 0.14
Enrollment weight (proxy for birthweight)	0.04 (−0.06, 0.14) 0.41	0.13 (0.04, 0.23) 0.01	0.09 (−0.01, 0.19) 0.06
Maternal height	0.01 (−0.003, 0.03) 0.12	0.02 (0.01, 0.04) 0.006	0.02 (0.002, 0.03) 0.03
Maternal education	0.03 (−0.01, 0.06) 0.11	0.05 (0.02, 0.09) 0.003	0.04 (0.01, 0.08) 0.02
WAMI (socioeconomic status)	1.13 (0.24, 2.03) 0.01	1.70 (0.81, 2.55) 0.0002	1.20 (0.31, 2.09) 0.01
Income (/$100)	0.08 (0.03, 0.12) 0.001	0.07 (0.02, 0.11) 0.004	0.05 (0.01, 0.10) 0.02
Percent days exclusively breastfed	0.07 (−0.40, 0.54) 0.76	0.41 (−0.05, 0.87) 0.08	−0.17 (−0.63, 0.28) 0.46
Sex (female versus male)	0.10 (−0.09, 0.29) 0.30	−0.23 (−0.41, −0.05) 0.01	0.08 (−0.1, 0.27) 0.39
2 year-old height	0.17 (0.08, 0.27) 0.0003	0.17 (0.08, 0.26) 0.0002	0.14 (0.05, 0.24) 0.002
5 year-old height	0.19 (0.09, 0.29) 0.0002	0.20 (0.1, 0.30) <0.0001	0.21 (0.11, 0.31) <0.0001
Height at assessment (∼8 year-old height)	0.22 (0.12, 0.31) <0.0001	0.27 (0.17, 0.36) <0.0001	0.20 (0.11, 0.29) <0.0001

CL = confidence limits; WAMI = Water/Sanitation, Assets, Maternal education, and Income. Models adjusted for study site and age.

## DISCUSSION

This study assessed longer-term follow-up cognitive testing at school age in a longitudinal birth cohort to examine relationships between early-life enteric pathogen exposures and school-age cognitive outcomes. Bacterial and protozoal pathogens showed small negative associations with cognitive outcomes, although the estimates were imprecise. The research sample may not be large enough to detect small changes; however, even when controlling for many variables that are associated with school-age outcomes, these results are consistent with prior work on the impact of early-life enteric infections on longer-term morbidity. For example, we found that bacterial and protozoal infections were associated with linear growth decrements at 2 and 5 years of age in MAL-ED, but viral infections were not.^5^ Bacterial and protozoal pathogens may have larger effects on the gut than viruses and contribute to environmental enteric dysfunction, leading to long-term impacts on growth and cognition. Specifically, *Shigella* and *Campylobacter* are inflammatory pathogens, and both intestinal and systemic inflammation may be associated with poorer cognitive function.

It is potentially interesting that early-life *Cryptosporidium* and *Shigella* infections tended to associate with the lowest semantic fluency and reasoning (Figure 2). In a companion paper focused on *Shigella* infections and inflammation^24^, we demonstrate that the association of early-life *Shigella* prevalence with lower height-for-age-z-score at school age is consistent across the three sites and estimate site-specific associations of *Shigella* burdens with modest but consistently lower cognitive outcomes (reasoning and semantic and verbal fluency) in Brazil and Tanzania, but not South Africa. Hence, further work with rapid, simple fecal diagnostics for selected pathogens or inflammation that could be done in field settings may enable research to focus on subgroups in certain settings that could benefit from targeted antimicrobial and nutritional therapy.

This study had the benefit of longitudinal follow-up of children from birth to 6–8 years of age, as well as frequent monthly collection of stools to characterize enteric pathogen burden in the first 24 months of life in three settings of malnutrition and enteric disease. One limitation of this data is that by 6 to 8 years of age, many of the children in the research site in Tanzania still had not had much formal schooling, and thus the second executive function task of naming words that begin with certain letters was more challenging for them. Children in all three sites in settings of malnutrition, enteric disease, and low resources were more likely to be below age expectancy for these cognitive assessments.

Our study relied on the lessons from the previous work of the cognitive assessment team of the MAL-ED study.^21^ We found that it is difficult to measure cognitive development across languages and cultures. However, by using assessments that are less reliant on specific culture and language and that have been piloted with children in the area, a distribution of results can be obtained. This study only measured three areas of childhood cognitive abilities: semantic fluency, phonemic fluency, and reasoning skills. Although these are important areas of functioning, other studies may measure other important areas of childhood cognition to continue to examine the role of early enteric pathogen exposure in child development.

There are many proximal factors to school-age cognition and learning, and the ones that are likely most influential early in life, such as maternal education level and family resources, were found to have stronger associations with higher cognitive scores in this study. These results suggest that interventions directly on cognitive development, such as cognitive stimulation, may have larger impacts on cognitive development. Child growth was also associated closely with cognitive scores, highlighting the importance of nutrition. However, there may also be a small role of interventions for early-life enteric pathogens to improve cognitive abilities in school-age children.
